# Crash Landing of Thyroid Storm: A Case Report and Review of the Role of Extra-Corporeal Systems

**DOI:** 10.3389/fendo.2021.725559

**Published:** 2021-08-20

**Authors:** Shir Lynn Lim, Kangjie Wang, Pak Ling Lui, Kollengode Ramanathan, Samantha Peiling Yang

**Affiliations:** ^1^Department of Cardiology, National University Heart Center, Singapore, Singapore; ^2^Division of Endocrinology, Department of Medicine, National University Hospital, Singapore, Singapore; ^3^Department of Hematology-Oncology, National University Cancer Institute, Singapore, Singapore; ^4^Cardiothoracic Intensive Care Unit, National University Heart Center, Singapore, Singapore; ^5^Department of Surgery, Yong Loo Lin School of Medicine, Singapore, Singapore; ^6^Department of Medicine, Yong Loo Lin School of Medicine, Singapore, Singapore

**Keywords:** thyroid storm, multi-organ failure, extra-corporeal membrane oxygenation, therapeutic plasma exchange, continuous renal replacement therapy, out-of-hospital cardiac arrest

## Abstract

Thyroid storm is a rare but life-threatening endocrinological emergency with significant mortality ranging from 10-30% with multi-organ involvement and failure. In view of the rarity of this condition and efficacy of established first line medical treatment, use of extra-corporeal treatments are uncommon, not well-studied, and its available evidence exists only from case reports and case series. We describe a 28-year-old man who presented with an out-of-hospital cardiac arrest secondary to thyroid storm. Despite conventional first-line pharmacotherapy, he developed cardiogenic shock and circulatory collapse with intravenous esmolol infusion, as well as multi-organ failure. He required therapeutic plasma exchange, concurrent renal replacement therapy, and veno-arterial extra-corporeal membrane oxygenation, one of the few reported cases in the literature. While there was clinical stabilization and improvement in tri-iodothyronine levels on three extra-corporeal systems, he suffered irreversible hypoxic-ischemic brain injury. We reviewed the use of early therapeutic plasma exchange and extra-corporeal membrane oxygenation, as well as the development of other novel extra-corporeal modalities when conventional pharmacotherapy is unsuccessful or contraindicated. This case also highlights the complexities in the management of thyroid storm, calling for caution with beta-blockade use in thyrocardiac disease, with close monitoring and prompt organ support.

## Introduction

Thyroid storm (TS) is a life-threatening exacerbation of the hyperthyroid state characterized by multi-organ dysfunction of the cardiovascular, thermoregulatory, gastrointestinal-hepatic and central nervous systems. While the incidence among hospitalized patients is estimated to be low at 1-2% (1), the overall mortality is 10-30% (2), with a 12-fold increase in mortality compared to individuals with thyrotoxicosis (3). The diagnosis of TS is additionally challenging due to the absence of specific clinical or laboratory findings. Early recognition of this condition is key, as it allows for prompt and specific treatment, as well as early identification of organ dysfunction with initiation of supportive measures in the intensive care setting if required.

We present a case of TS in a young patient with undiagnosed Graves’ disease, presenting with an out-of-hospital cardiac arrest. Initially hemodynamically stable following return of spontaneous circulation, he developed circulatory collapse after intravenous esmolol infusion, initiated for control of tachycardia. There was consequent multi-organ failure which contraindicated the use of standard anti-thyroid drug therapy. He required three extra-corporeal systems of continuous renal replacement therapy (CRRT), veno-arterial extra-corporeal membrane oxygenation (VA-ECMO) and therapeutic plasma exchange (TPE) for stabilization, one of the few reported cases in the literature.

## Case Description

A 28-year-old male presented with an out-of-hospital ventricular fibrillation (VF) arrest, preceded by an acute respiratory illness. There was return of spontaneous circulation after 60 minutes of resuscitation with bystander cardiopulmonary resuscitation and external defibrillation by paramedics. In the Emergency Department, he was febrile at 40.5 degrees Celsius, hypertensive with a blood pressure of 146/83mmHg and tachycardic with a heart rate of 155 beats per minute. Physical examination was unremarkable, except for a Glasgow Coma Scale of 3. No goiter was seen on examination. Corroborative history from his family confirmed symptoms of heat intolerance, loss of weight, hand tremors and palpitations in the preceding two months, as well as a maternal history of Graves’ thyrotoxicosis.

**Figure 1:**
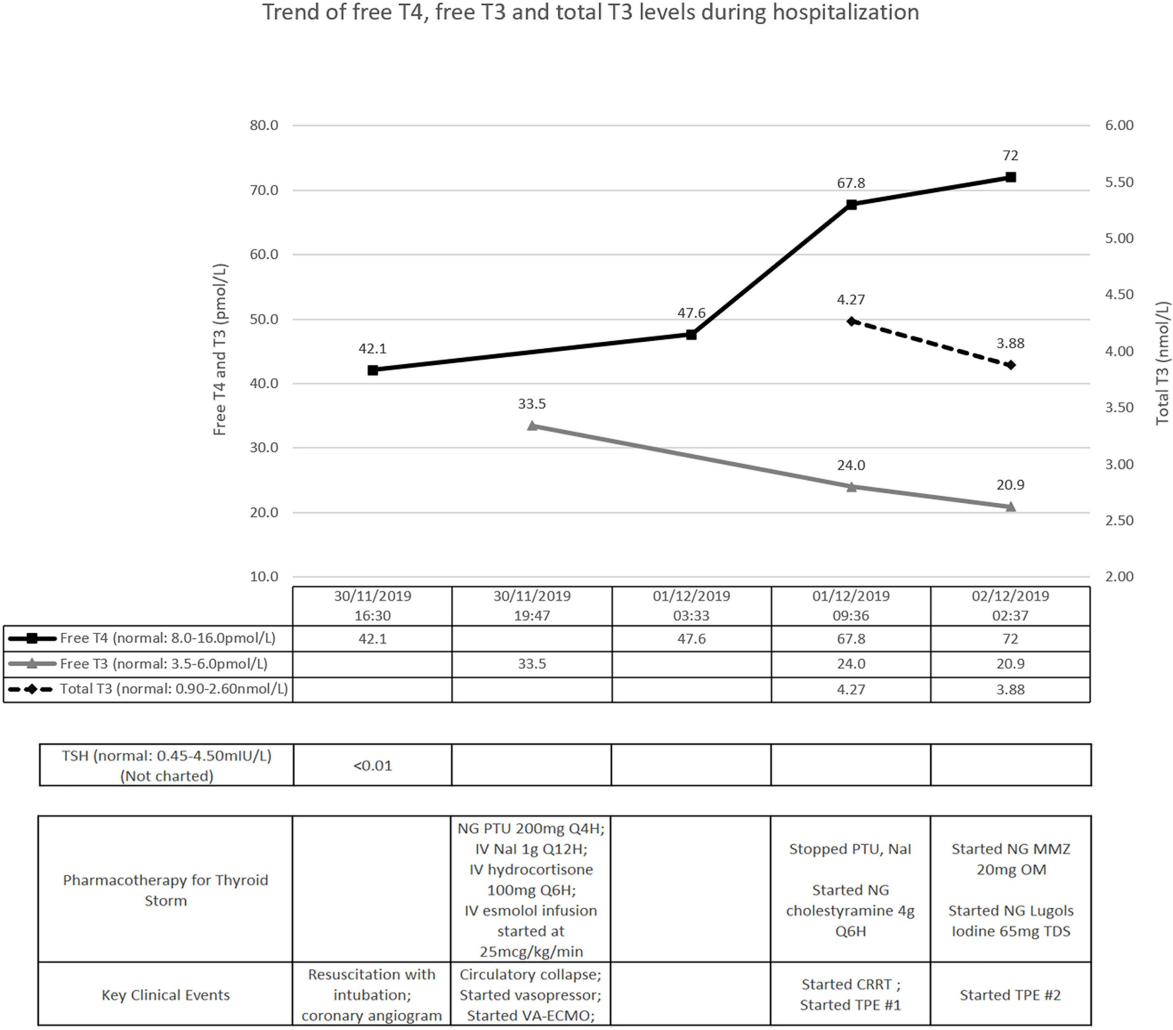
Timeline of key clinical events. T4, thyroxine; T3, tri-iodothyronine; TSH, thyroid-stimulating hormone; NG, nasogastric; IV, intravenous; PTU, propylthiouracil; NaI, sodium iodide; VA-ECMO, veno-arterial extra-corporeal membrane oxygenation; CRRT, continuous renal replacement therapy; TPE, therapeutic plasma exchange; MMZ, methimazole.

Initial investigations showed elevated inflammatory markers, mixed respiratory and metabolic acidosis, raised troponin I, but with normal electrolyte levels. Electrocardiogram confirmed sinus tachycardia. Chest radiograph showed prominent pulmonary vasculature without evidence of pneumonia. Point-of-care echocardiogram showed impaired left ventricular systolic function without other obvious abnormalities; the marked sinus tachycardia precluded accurate estimation of the left ventricular ejection fraction (LVEF). Computed tomographic (CT) scan of the brain was normal, and urine drug screen was negative. A coronary angiogram performed was normal, and a provisional diagnosis of acute myocarditis was made. Thyroid function test, sent as part of investigations for myocarditis, showed thyrotoxicosis with an elevated serum free thyroxine (FT4) level of 42.1pmol/L (reference range: 8.0-16.0pmol/L) and a suppressed serum thyroid stimulating hormone (TSH) at <0.01mIU/L (reference range: 0.45-4.50mIU/L) (**Figure 1**) – our patient had thyroid storm complicated by thyrocardiac disease, with a Burch-Wartofsky score of 105. His thyroid-stimulating hormone receptor antibody eventually returned elevated at >40IU/L (normal ≤2.0IU/L), confirming underlying Graves’ disease.

**Figure 2:**
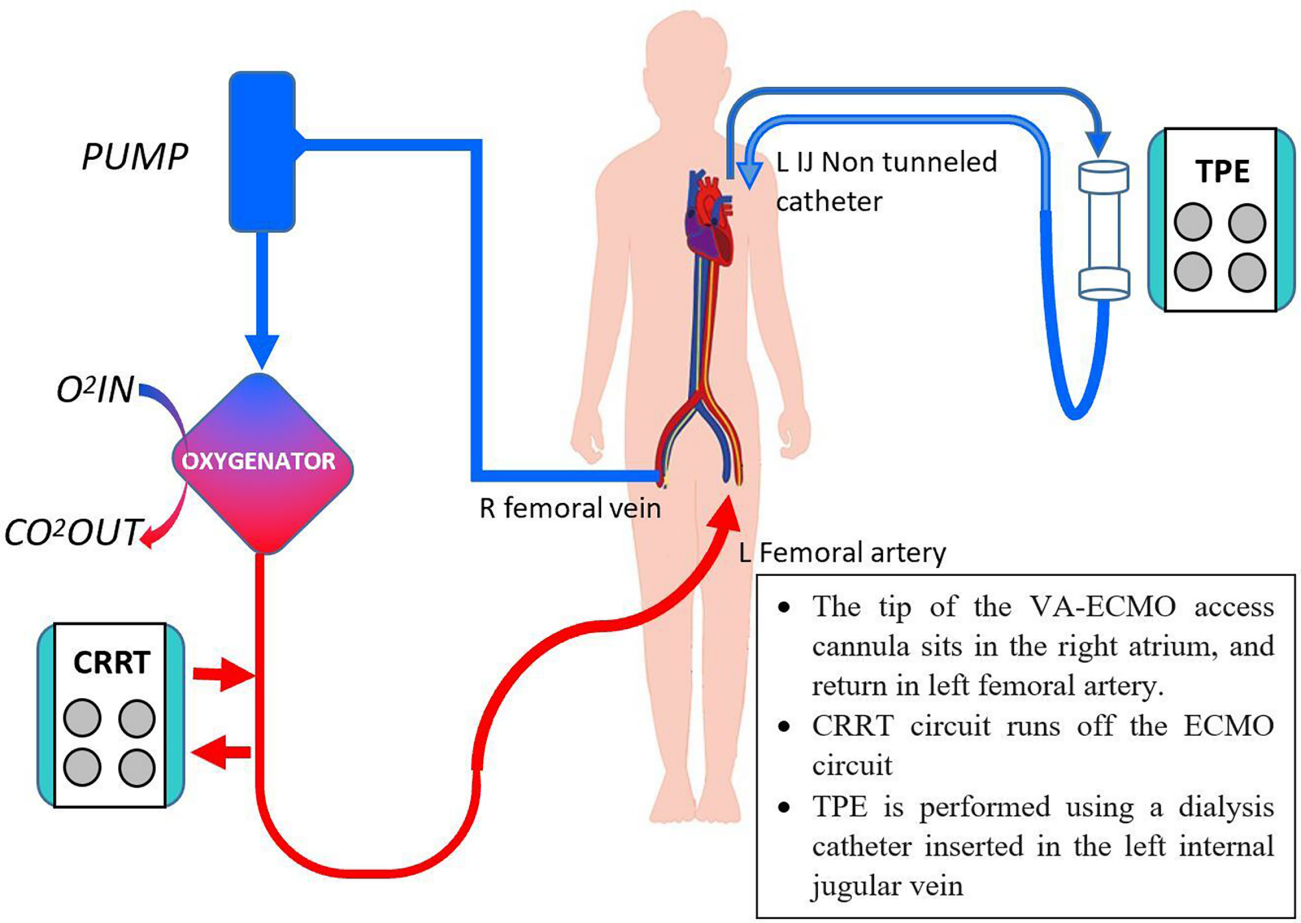
Concurrent CRRT, VA-ECMO and TPE. O_2_, oxygen, CO_2_, carbon dioxide, others as per **Figure 1**.

Treatment was promptly initiated with nasogastric propylthiouracil, intravenous sodium iodide and hydrocortisone (**Figure 1**). Temperature was controlled with a cooling blanket. Judicious low dose esmolol infusion was commenced at 25mcg/kg/min to manage the tachycardia. This was followed shortly by a pulseless electrical activity arrest. Despite a short downtime of three minutes and prompt cessation of beta-blockade, he required high doses of noradrenaline and vasopressin thereafter. He remained persistently hypotensive with maximal dual vasopressor support, and was initiated on VA-ECMO support (**Figure 2**).

Further investigations revealed worsening transaminitis and anuric acute kidney injury, requiring CRRT. Transthoracic echocardiogram showed severe left ventricular systolic dysfunction with estimated LVEF of 10%. Pharmacological options were now limited to cholestyramine and hydrocortisone. We decided to institute TPE; with the first cycle performed on day 2 of admission, with 2.5L of albumin and 0.5L of saline (**Figure 1**). After the first cycle, his vasopressor support reduced significantly (only requiring low dose noradrenaline infusion) and his tri-iodothyronine (T3) levels improved (**Figure 1**). As his FT4 continued to worsen, nasogastric methimazole and Lugol’s iodine were cautiously started, along with second TPE cycle, on the third day.

His pupils were noted to be fixed and dilated with the second cycle of TPE ongoing. Urgent CT scan of the brain revealed diffuse cerebral edema with severe mass effect and tonsillar herniation. A decision was made in conjunction with his family for withdrawal of care, given the grave prognosis.

## Discussion

### Evidence of Use of Extra-Corporeal Systems in TS

Our patient is one of the few reported cases where multiple extra-corporeal systems were used (TPE, CRRT, and VA-ECMO) in TS management. On review of the medical literature of articles in English, there has only been four case reports describing the concurrent use of TPE and ECMO in patients with thyrotoxicosis with circulatory collapse (4–7), with one of them reporting the use of three extra-corporeal systems (4).

Characterized by extreme multi-systemic manifestations of thyrotoxicosis, TS is uncommon but potentially fatal, with a mortality rate of 10-30% (2). Standard first-line pharmacotherapy in TS aims to block production and release of thyroid hormones with propylthiouracil or methimazole, inhibit release of pre-formed thyroid hormones with iodine, decrease peripheral conversion of T4 to T3 with propylthiouracil and steroids, and treat adrenergic symptoms with beta-blockade, prior to definitive treatment in the form of surgery or radioactive iodine (RAI) ablation. These methods are efficacious and rapid acting. However, certain patients are not able to tolerate, or fail pharmacotherapy, as seen in our patient with multi-organ failure precluding the use of conventional treatment. In such cases, alternative treatment including use of extra-corporeal systems must be considered, of which TPE is the most well established.

TPE is an extra-corporeal blood purification technique used for eliminating large molecular substances. Currently, TPE has a Class II indication for TS in the 2019 American Society for Apheresis (ASFA) guidelines, either as a standalone or adjunct therapy, although the grade 2C level of recommendation suggests the evidence arises mostly from case reports and case series, with an absence of prospective randomized controlled trials (8). TPE is postulated to work by several mechanisms including: 1) reducing protein-bound thyroid hormones of which 99.97% of total serum T4 and 99.7% of total serum T3 are bound to plasma proteins thyroxine binding globulin (TBG), transthyretin and albumin (9), 2) additionally reducing autoantibodies and cytokines of a predominantly Th1 pattern including interleukin-2, interferon-γ and tumor necrosis factor-α (10–12), and 3) removing 5’-monodeiodinase which converts T4 to T3 (12). TPE is shown to reduce all of free and total T4 and T3 (13), by an estimated 10-80% (12), and at a greater rate than standard medical therapy for patients with hyperthyroidism (14). However, these effects are transient and usually last for only 24-48 hours, with a potential risk for rebound thyrotoxicosis (12). This appears related to the fact that only thyroid hormones from the intravascular compartment is removed, with rapid re-equilibrium from the extravascular spaces. A study in 13 healthy individuals showed the intravascular component of thyroxine accounts for only 26% of the distribution (15), with the other sites of distribution found to be 14% in the liver, 34% in extrahepatic tissue and 26% in extracellular fluid pools. These transient effects suggest that multiple cycles of TPE may be required, and should be used only as a temporizing measure for definitive treatment (12). TPE can be performed with either plasma or albumin replacement, and the ASFA guideline does not preferentially recommend the use of plasma or albumin. It has been proposed that plasma has the theoretical advantage of containing binding proteins TBG and transthyretin, which has higher affinity to bind free T4 and T3, as well as avoid depleting coagulation factors thus avoiding complications of bleeding (13). However, there is similarly a theoretical risk of the presence of thyroid hormones from donor plasma, as well as higher risks of transfusions reactions and infections with use of plasma. Till date, there are no direct head-to-head trials between the use of plasma and albumin in TPE in patients with TS. TPE is generally well tolerated, with risks of minor side effects ranging from about 5% (9) to 36% (16), including nausea and vomiting, vagal or hypotensive response and transfusion reactions. The risk of death with TPE for any indication is exceedingly rare at 0.05% (17), and this is usually attributed to the severity of the underlying condition.

ECMO is an established life-saving treatment option for patients who develop acute cardiopulmonary failure (18), although its use in endocrinological emergencies is still under research (19). Use of ECMO in patients with thyrotoxicosis have largely been reported in the form of case reports or case series within the literature. In 2011, Hsu et al. (20) first reported a series with the use of supportive ECMO ranging from 19-114 hours in four cases of thyrotoxicosis-induced cardiovascular collapse. Three patients survived, with normalization of thyroid function and improved cardiovascular function. A subsequent review by White et al. (21) published in 2018 reported the successful use of ECMO with survival in 11 out of 14 patients (22–24) between 1970 to 2017 with thyrotoxicosis-induced cardiomyopathy, with near complete recovery of left ventricular function. A review of the cases with the use of ECMO and other extra-corporeal systems in patients with severe thyrotoxicosis or thyroid storm has been summarized in **Table 1**, including additional new cases reported from 2018 to 2021 (25–28) and conference poster reports (29–32). These 27 cases (inclusive of our case) showed survival in 85.2% (23 of the 27 cases), with survival in all four of the reported cases requiring additional extra-corporeal support of TPE or CRRT (4–7). ECMO was initiated for either cardiovascular collapse or circulatory shock, and lasted between 19 hours to 18 days. Within the reports of successful outcomes, all cases reported clinical and biochemical improvement in thyrotoxicosis, as well as improvement in cardiac function, although numerical data were not available in some of the reports. The details of these cases are reported in **Table 1**. The use of ECMO however, must be weighed against the contraindications and complications of ECMO use, including bleeding, thromboembolism, strokes and access injuries such as hemorrhage, arterial dissection, and distal limb ischemia (18).

**Table 1:** Summary of cases with use of ECMO and other extra-corporeal systems in patients with severe thyrotoxicosis or thyroid storm.

Study	Patient gender	Patient age	Indication for ECMO	Duration of ECMO	Pre-ECMO LVEF	Post-ECMO LVEF	Other extra-corporeal system used	Biochemical response after extra-corporeal treatment	Outcome
Koh et al. (4)	Male	44	PEA collapse with shock	~3 days	20-25%	–	TPE (4 cycles), CRRT	fT4 from 57 to 22pmol/L; fT3 from 12.4 to 6.0pmol/L	Survived. Underwent thyroidectomy.
Wong et al. (5)	Male	44	Recurrent PEA collapse	3 days	20%	35%	TPE (3 days)	fT4 from 61.3 to 22.0pmol/L; fT3 from 23.5 to 4.3pmol/L	Survived. Underwent thyroidectomy.
Eyadiel et al. (6)	Female	27	Cardiogenic shock	6 days	<10%	Almost completely recovered*	TPE (3 cycles)	Normalization of T3*	Survived.
Manuel et al. (7)	Male	26	PEA collapse with shock	24 hours	–	*-*	TPE (2 cycles) *via* ECMO circuit	fT4 >100pmol/L at baseline, reportedly improved on discharge*	Survived. Underwent thyroidectomy.
Chao et al. (19)	Male	47	Refractory shock	19-115.6 hours (mean 82 hours)	20-40% (Mean 24%)	38-64% (mean 55%) on day 6	–	–	Expired, from multi-organ failure
	Male	43						–	Expired, from hepatic failure
	Female	37						fT4 54.8-308.9pmol/L at baseline, reportedly improved on discharge*	Survived
	Male	42							Survived
	Female	33							Survived
Hsu et al. (20)	Male	47	Cardiogenic shock	19 hours	32%	–	–	–	Expired
	Male	43	PEA collapse	114 hours	20%	64%		fT4 31.1pmol/L at baseline; serum T3 3.64 improved to 1.69nmol/L	Survived
	Female	37	PEA collapse	94 hours	32%	60%		fT4 96.5 improved to 19.3pmol/L	Survived
	Male	42	Shock	102 hours	29%	58%		fT4 57.9 improved to 18.3pmol/L	Survived
White et al. (21)	Female	57	PEA collapse	10 days	<10%	20-30%	–	Clinical improvement. Improvements in thyroid hormone not documented*	Survived
Pong et al. (22)	Male	33	Cardiogenic shock	4 days	10%	51%	–	fT4 55pmol/L, normalized after 1 week	Survived
	Female	35	Cardiogenic shock	4 days	17%	52%	–	fT4 44pmol/L, normalized after 4 days	Survived
Allencheril et al. (23)	Male	29	PEA collapse	7 days	<20%	45-49%	–	Clinical improvement. Improvements in thyroid hormone not documented*	Survived
Kiriyama et al. (24)	Female	54	Cardiogenic shock	18 days	<20%	Almost completely recovered*	–	fT4 49.3pmol/L, fT3 7.04pmol/L at baseline, reportedly improved on discharge*	Survived
Kim et al. (25)	Male	52	Cardiogenic shock	6 days	<20%	40%	–	fT4 100.0pmol/L, fT3 7.04pmol/L at baseline, reportedly improved on discharge*	Survived
Genev et al. (26)	Female	37	Cardiogenic shock	8 days	30%	35%	–	fT4 from 60.5 to 12.9pmol/L; fT3 from 13.6 to 2.5pmol/L	Survived
Voll et al. (27)	Female	35	Recurrent PEA collapse with shock	3 days	<20%	Normalized*	–	fT4 79pmol/L, fT3 47pmol/L, reportedly improved on discharge*	Survived. Underwent thyroidectomy.
Chao et al. (28)	Female	35	PEA collapse	65 hours	5%	65%	–	fT4 100.8pmol/L, fT3 16.3pmol/L, reportedly improved on discharge*	Survived
Al-Saadi et al. (29)	Male	29	Cardiac arrest	6 days	<20%	–	–	fT4 83.5pmol/L, fT3 7.04pmol/L at baseline, reportedly improved on discharge*	Survived
Kauth et al. (30)	Male	53	PEA collapse	12 days	–	Normalized*	–	fT4 66.2pmol/L, fT3 19.2pmol/L, normalized on discharge*	Survived
Karahalios et al. (31)	Female	29	PEA collapse	2 weeks	Biventricular failure*	Improved*	–	*fT4 79.8pmol/L at baseline*	Survived
Starobin et al. (32)	Male	33	Cardiogenic shock	–	10%	Normalized*	–	–	Survived

‘*’ denotes incomplete data from articles, while ‘-’ denotes absence of reported data.

Novel therapies are also increasingly considered for TS, using principles similar to TPE by removing protein bound thyroid hormones. Case reports with the use of dialysis has been proposed in management of thyroid storm, most notably with CRRT, which is preferred due to its better tolerability in hemodynamically unstable patients due to its slower rate of exchange of fluids and solutes. Parikh et al. (33) and Koball et al. (34) illustrated the sequential use of single pass continuous veno-venous albumin dialysis after limited response to TPE, demonstrating a more sustained improvement in thyroid hormones with less rebound thyrotoxicosis, as well as greater removal of thyroid hormones overall. Other studies have shown the additive effects of TPE and CRRT in removal of thyroid hormones (35), while another study reported a correlation of improvement of total T3 and free T4 levels of up to 80% with concomitant CRRT and standard medical therapy (without TPE), although the exact mechanisms are unclear (36). The Molecular Adsorption Recirculation Systems (MARS) has also been used, with one case report with TS and severe liver dysfunction showing rapid resolution of thyroid hormones and improvement of bilirubin (37). A retrospective case series also demonstrated significant improvement in thyroid hormone levels in patients with hyperthyroidism with severe liver dysfunction (although this study was primarily powered to show improvement and safety of use of RAI with combined with MARS in patients with severe hyperthyroidism and liver disease) (38). These reports provide early evidence of the utility of novel extra-corporeal systems in correcting thyroid hormone levels especially in patients with either kidney or liver dysfunction, although more research into the underlying mechanism and validation of results are required before recommendations can be made for its supportive use.

These cases provide some evidence of the use and benefits of extra-corporeal systems in the management of TS, after conventional pharmacotherapy is unsuccessful or contraindicated. Owing to the efficacy of pharmacotherapy and risks of extra-corporeal systems, conventional pharmacotherapy should be always be instituted as initial therapy. Comparison trials between pharmacotherapy and extra-corporeal systems or randomized controlled trials are unavailable due to the rarity of TS, and are unlikely to be performed now given the established efficacy of first line pharmacological agents. Retrospective analysis from the National Inpatient Database in Japan has shown that use of extra-corporeal systems is associated with higher mortality. It reported increased mortality in patients requiring hemodialysis and TPE with adjusted odds ratio for mortality at 4.81. The mortality was 61.9% in 13 out of 21 patients, compared to a mortality of 43.3% requiring either hemodialysis or TPE, and 7.8% requiring neither support. The use of ECMO had a trend towards increased mortality (2.86, CI 0.69-11.92), with a mortality of 72.2% among 13 of 18 patients, as compared to 9.3% in patients not requiring ECMO (39). These numbers, albeit small, suggest a significantly higher mortality in patients requiring use of extra-corporeal systems, and this differs from the established mortality rate of 10-30%, and vary significantly from the numbers in our review and White et al.’s review in patients requiring ECMO (21). Similarly Muller et al. (12) showed the use of TPE showed significant clinical and biochemical improvement. As patients requiring extra-corporeal systems are typically patients who are more critically ill and have multi-organ failure, as well as the possibility of publication bias, it is likely that the true survival rate of these patients in thyroid storm treated with extra-corporeal systems is likely lower than the published literature. Further research, possibly in the form of prospective multinational studies, may be required in view of the small numbers and limited data currently.

### Beta-Blockade – A Double-Edged Sword in TS

While our patient received guideline-directed TS pharmacological therapy in a timely fashion, the development of circulatory collapse with consequent multi-organ failure following intravenous esmolol infusion, an ultra-short acting beta-blocker, deserves further discussion.

The cardiovascular effects in TS are driven largely by T3, leading to increased chronotropy and inotropy, improved diastolic relaxation and decreased peripheral resistance, eventually resulting in high cardiac output (CO) heart failure (HF), estimated to be seen in 6% of patients with thyrotoxicosis. This is thought to be reversible with treatment with thyrotoxicosis, with a small study showing improving in LVEF from 28% to 55% (40). Cardiomyopathy and LV dysfunction, on the other hand, are only seen in 1% (20, 41). HF with low CO has been reported with prolonged severe hyperthyroidism, consequent to persistent tachycardia, and pathologic increase in cardiac workload with demand-supply mismatch (42). Aside from cardiomyopathy, there is an increased risk of arrhythmias with thyrotoxicosis, typically supraventricular, with rare reported cases of thyrotoxicosis-related VF related to congenital coronary anomalies, hypokalemia, coronary vasospasm and early repolarization (43), none of which were present in our patient. It is plausible that our patient had low CO thyrocardiac disease with an additional component of myocardial stunning post-cardiac arrest, but his stormy course precluded detailed cardiac imaging.

Tachycardia is almost always present in TS, and patients with tachycardia exceeding 150 beats per minute are associated with a higher mortality rate in a retrospective Japanese cohort (44). Accordingly, the Japanese Thyroid Association and Japanese Endocrine Society 2016 guidelines (45) recommend aggressive control of tachycardia including the use of ultra-short acting beta-blockers including esmolol or landiolol. New data are emerging which support the use of esmolol over propranolol, due to its shorter half-life elimination (nine minutes, *versus* 2.3 hours respectively) and duration of action, as well as its relatively higher beta 1-selectivity (46). The comparative use of esmolol and propranolol has been studied in other populations such as patients with supraventricular tachycardia, which showed similar response rate but more adverse effect of hypotension seen in the esmolol group (45%, as compared to 18%), although these were mostly asymptomatic and resolved quickly with no complications (47). Regardless of choice of beta-blockers, its use must be considered with caution in patients with decompensated HF or other features of low CO, where the thyroid-induced hyperadrenergic state plays an important compensatory role in maintaining CO. This is related to either direct catecholamine action or an interaction between the adrenergic system and excessive circulating thyroid hormone (48). The abolishment of that sympathetic drive through the use of beta-blockers is postulated to lead to the circulatory collapse, as seen in our case. Though initially hypertensive, the temporal association of esmolol infusion and PEA arrest led us to conclude it caused or at least triggered the hemodynamic decompensation in our patient. Abubaker et al. (49) reviewed a total of 11 cases of circulatory collapse with the use of beta-blockade, mostly with long acting agents including bisoprolol, metoprolol, propranolol, with all but one patient showing evidence of underlying heart failure or cardiomyopathy. The author also highlighted the challenges in managing uncontrolled tachycardia in these patients, with two cases eventually requiring esmolol and landiolol use. To date, there has been no head-to-head trials between the longer acting propranolol as compared to the ultra-short acting esmolol or landiolol. There has no reports of circulatory collapse with use of intravenous esmolol, and only one case report with landiolol (50). Despite the use of ultra-short acting esmolol, circulatory collapse in our case underscores its class effect, and is strongly associated with fatal outcomes in TS. Close cardiac monitoring and prompt institution of VA ECMO support, as what was done in this case, are recommended. Other forms of supportive therapy including CRRT may be considered until effective and definitive therapies can be instituted to treat TS.

## Conclusion

We highlight a case of TS presenting with out-of-hospital cardiac arrest, with further hemodynamic decompensation following beta-blockade and multi-organ failure which limited therapeutic options. Despite prompt initiation of CRRT, VA-ECMO and TPE, he sustained hypoxic-ischemic brain injury. Underscoring the complexities in TS, this case calls for caution with beta-blockade in thyrocardiac disease, close monitoring and prompt organ support, and consideration of early TPE when conventional options fail. A review of the use of TPE and other extra-corporeal systems shows that TPE may be an underutilized rescue treatment for severe thyroid storm not amenable to conventional pharmacotherapy or contraindicated due to side effects or multi-organ involvement. Further study of novel extra-corporeal therapies for TS is needed to uncover its therapeutic potential, especially in the Intensive Care setting.

## Author Contributions

All authors were involved in the management of the patient. SL, KW, and SY wrote the first draft of the manuscript. PL and KR reviewed and edited the manuscript. All authors contributed to the article and approved the submitted version.

## Conflict of Interest

The authors declare that the research was conducted in the absence of any commercial or financial relationships that could be construed as a potential conflict of interest.

## Publisher’s Note

All claims expressed in this article are solely those of the authors and do not necessarily represent those of their affiliated organizations, or those of the publisher, the editors and the reviewers. Any product that may be evaluated in this article, or claim that may be made by its manufacturer, is not guaranteed or endorsed by the publisher.
